# Determining the optimal model for role-substitution in NHS dental services in the United Kingdom

**DOI:** 10.1186/1472-6831-13-46

**Published:** 2013-09-24

**Authors:** Paul Brocklehurst, Stephen Birch, Ruth McDonald, Martin Tickle

**Affiliations:** 1 School of Dentistry, The University of Manchester, Oxford Road, Manchester, UK; 2 School of Community Based Medicine, The University of Manchester, Oxford Road, Manchester, UK; 3 Warwick Business School, University of Warwick, Coventry, UK

## Abstract

**Background:**

Role-substitution describes a model of dental care where Dental Care Professionals (DCPs) provide some of the clinical activity previously undertaken by General Dental Practitioners. This has the potential to increase technical efficiency, the capacity to care and reduce costs. Technical efficiency is defined as the production of the maximum amount of output from a given amount of input so that the service operates at the production frontier i.e. optimal level of productivity. Academic research into technical efficiency is becoming increasingly utilised in health care, although no studies have investigated the efficiency of NHS dentistry or role-substitution in high-street dental practices. The aim of this study is to examine the barriers and enablers that exist for role-substitution in general dental practices in the NHS and to determine the most technically efficient model for role-substitution.

**Methods/design:**

A screening questionnaire will be sent to DCPs to determine the type and location of role-substitutive models employed in NHS dental practices in the United Kingdom (UK). Semi-structured interviews will then be conducted with practice owners, DCPs and patients at selected sites identified by the questionnaire. Detail will be recorded about the organisational structure of the dental team, the number of NHS hours worked and the clinical activity undertaken. The interviews will continue until saturation and will record the views and attitudes of the members of the dental team. Final numbers of interviews will be determined by saturation.

The second work-stream will examine the technical efficiency of the selected practices using Data Envelopment Analysis and Stochastic Frontier Modeling. The former is a non-parametric technique and is considered to be a highly flexible approach for applied health applications. The latter is parametric and is based on frontier regression models that estimate a conventional cost function.

**Discussion:**

Maximising health for a given level and mix of resources is an ethical imperative for health service planners. This study will determine the technical efficiency of role-substitution and so address one of the key recommendations of the Independent Review of NHS dentistry in England.

## Background

Maximising health for a given level and mix of resources is an ethical imperative for health service planners [1]. In 2009, the Independent Review of National Health Service (NHS) dentistry in England concluded that there was an overwhelming need to make best use of the whole dental workforce [2]. As a result, the Department of Health (DH) began piloting a new NHS dental contract in 2010, with an "emphasis on prevention while meeting patients’ treatment needs more effectively" [3].

Designing a workforce that is appropriate for the future requirements of the NHS is imperative and Birch argues that four underlying determinants are key: size of the population, the level of disease (or need for care) in this population, the level of service required to address this need and the technical efficiency of the workforce [4]. Whilst the former two lie outside of the control of health service planners, the latter are subject to change and one aspect of technical efficiency (productivity) that is under-utilised in dentistry is role-substitution. Role-substitution describes a model of care in which Dental Care Professionals (DCPs) provide the clinical activity previously undertaken by General Dental Practitioners (GDPs) [5]. The innovative use of this model has the potential to increase technical efficiency, increase the capacity to care and reduce service costs.

Technical efficiency is defined as the production of the maximum amount of output from a given amount of input [6] so that the service operates at the production frontier i.e. optimal level of productivity. Academic research into technical efficiency is becoming increasingly utilised in health care [7], although no studies have investigated the efficiency of NHS dentistry or examined the impact of role-substitution in high-street dental practices. Instead, NHS dental service provision has developed historically, with levels of future service provision being determined by past levels of activity, at a time when disease levels at a population level are reducing [8]. This mismatch between service provision and disease experience has been allowed to continue and is influenced by a number of factors including the political influence of the profession, a conflation between dental need and demand for dental services and supplier induced demand [9]. Given the projected improvement in the oral health of the population [8], this disparity between the level of service provision and level of dental need is likely to deteriorate further. If this issue is not addressed, NHS resources could continue to be devoted to the use of highly paid and skilled providers to perform relatively simple tasks on an increasingly healthy practice population that less costly staff are competent to carry out.

Unlike their medical counterparts, who receive substantial support from the NHS for investment in both capital and labour expenditure, high-street dental practices operate as small independent discrete enterprises, where profitability and an adequate cash-flow are essential for survival. As a result, GDPs are very sensitive to incentives within the dental contract [10-12], although intrinsic motivation and professional standards can also be important moderators [13]. Retrospective fee-for-service systems have been shown to lead to over-treatment in order to maximize profit [9,11,14]. Prospective per-capita systems reduces the financial risk for the third party payer, but at the cost of patient-selection ("skimping" and "dumping") and under-treatment [15,16].

Empirical research from medicine suggests that appropriately trained nurses can deliver high quality care that matches medics in preventive health care, routine follow-up of patients with long term conditions and as the first contact for people with minor illness [17-19]. However, efficiency gains are only possible if doctors stop carrying out the tasks delegated to nurses and focus on tasks which only they can perform [20].

### Study aim

The aim of this study is to explore the barriers and enablers to role-substitution from the perspective of high-street GDPs, DCPs and patients. In addition, it aims to determine the technical efficiency of using role-substitution in high-street dental practices in the NHS in the United Kingdom.

### Study objectives

The objectives are to:

1. Conduct a cross-sectional study to determine the current working patterns of DCPs in NHS high-street dental practices across the UK.

2. To undertake semi-structured interviews with GDPs, DCPs and patients to explore barriers/enablers to the greater use of role-substitution; interviews will also be used to develop an understanding of the configuration of the dental team, collect the input data (NHS hours worked) and gain consent to collect the output data.

3. Collect the output data (clinical activity) from the relevant NHS contracting authority.

4. Use Data Envelopment Analysis (DEA) to identify the prevailing level of outputs that are produced by the inputs to determine the role-substitutive model that lies closest to the production possibilities frontier i.e. optimal service design.

5. Undertake Stochastic Frontier Modeling (SFM) to assess the external validity of DEA.

6. Examine how the technical efficiency of the different role-substitutive models varies across different retrospective and prospective payment systems for adults and children in the UK.

## Methods

The study received ethical approval from the North Wales Research Ethics Committee (Central & East) (REC - 12/WA/0403; IRAS - 114876).

### Work-stream One

#### ***Sample frame***

Different models of role-substitution will be identified by tracing DCP utilisation with an initial screening questionnaire sent to all members of the British Society of Dental Hygiene and Therapy. This will provide data on the working patterns and the extent and type of role-substitution used in different high-street dental practices working within the NHS. It will also enable the location of the different models to be mapped using ArcGIS software and the social deprivation of the area where the practice resides to be determined. NHS dental practices that utilise role-substitution will then be purposively sampled on the basis of the most commonly used models, taking account of the type of remuneration system employed, their geographic location and the level of deprivation.

#### ***Setting/context***

Following consent, a member of the research team will embed themselves in the selected high-street NHS dental practices.

#### ***Data collection***

Semi-structured interviews will be undertaken with both the principal GDP and the DCP from each NHS practice to determine the barriers and enablers to role-substitution. Accounts will not be automatically privileged and will be contrasted with observations made by the researcher, which will be recorded in situ.

The interviews will continue until saturation and will also record the organisational structure of the role-substitutive model employed and the number of NHS hours worked by the team (input data). Consent will also be sought to enable the relevant NHS contracting authority to be contacted for each practice in order to collect their levels of clinical activity (output data).

Patients will also be interviewed in each practice to determine the impact social acceptability could have on the organisation and efficiency of services that utilise role-substitution. Letters will be sent out to a random sample of adult patients who are due to attend when the GDP and DCPs are being interviewed. The letters, information sheets and consent forms will be sent two weeks in advance to enable an opportunity for the patients to ask any questions. Patients will be asked to return their consent forms prior to their appointment in the stamped addressed enveloped provided to ensure that an appointment with the interviewer can be made at the practice around the time of their dental appointment to minimize inconvenience.

#### ***Data analysis***

Data collection and analysis will run concurrently to facilitate constant comparative analysis. The initial coding frame will be developed from the first five interviews, depending on the number of themes identified. This will enable any potential issues to be identified at an early stage, which can then be discussed and reconciled. The recording from each interview will be transcribed verbatim and entered into NVivo on a personal computer. Thematic analysis will be undertaken in accordance with the recommendations of Braun & Clarke to develop a coding frame [21]:

1. The research team will immerse themselves in the data by reading and re-reading the transcriptions and noting down emerging ideas and patterns.

2. Initial codes will be generated by noting interesting features in the raw data in a systematic fashion across the entire data set.

3. The codes will then be collated into potential themes by looking for similarities and differences across the codes generated.

4. Themes will then be checked against the coded extracts and the raw data to ensure that they form a coherent pattern and are representative of what the participants were trying to convey.

5. The themes will then be examined to see how they form a coherent system of meaning and a thematic 'map’ of the codes will be generated showing their inter-relation.

6. Vivid and representative examples of each theme code and theme will be then selected that relate to the research question.

To facilitate triangulation, the transcripts will be read separately by the research team separately [22]. These will then be pooled and edited to produce the final version of the coding frame, with any disputes being resolved using a majority voting system.

As the results of Workstream Two become known, the thematic analysis will be re-examined to determine whether there are any systematic differences between the efficient, indifferent and inefficient practices identified.

### Work-stream Two

#### ***Theoretical framework***

DEA identifies the prevailing level of outputs that can be produced by a given level of inputs and so determines which substitutive model lies closest to the production possibilities frontier. It is a non-parametric technique that uses a linear-segmented efficiency frontier and a linear programming methodology [23,24]. It requires few assumptions to be satisfied and is considered to be a highly flexible approach that has been used in a range of pragmatic health applications [23,24]. Unlike parametric techniques, DEA can determine the relative efficiency of different models and can be undertaken without explicitly specifying the formal relations between inputs and outputs a priori [25].

SFM is parametric and is based on frontier regression models that estimate a conventional cost function. Residuals then form the measurement of efficiency and the error term is divided into a stochastic error term and a systematic inefficiency term. SFM has the advantage over DEA in that error is accounted for. However, there are a number of disadvantages: assumptions made about the inefficiency term in the model can be restrictive, the approach can confuse statistical noise with inefficiency; further analysis is sometimes required to separate the different components of the inefficiency term to determine technical efficiency [23,26,27]. As a result, Hollingsworth and Peacock recommend that the external validity of a technical efficiency model should be tested; this will be done by comparing efficiency assessments across both DEA and SFM using the same data [28]. This will also be triangulated with the results from Workstream One.

#### ***Sampling***

The input and output data collected from Workstream One will be used to undertake the DEA and SFM.

#### ***Data collection***

The organisational structure of the dental team and the number of NHS hours worked will be collected by Workstream One and will be used as the input data in the DEA and SFM. For the output data, intermediate measures of patient care will be utilized i.e. clinical activity rather than health gain. This is similar to the approach adopted in studies of technical efficiency in medicine, given that health outcomes are more difficult to determine over a short time frame and can be influenced by a number of factors that are external to the health care delivery system e.g. social deprivation [1].

#### ***Data analysis***

Efficiency in DEA is defined as the ratio of the weighted sum of outputs to its weighted sum of inputs [29]. The weights are specific to each unit so that 0 ≤ "role-substitutive model" ≤ 1 and a value of unity implies complete technical efficiency relative to the other models under scrutiny. Since the weights are not known a priori, they are calculated from the efficiency frontier by comparing one model with another [30]. DEA computes all possible sets of weights which satisfy all constraints and produces the highest efficiency score. This will be stated as a mathematical linear programming problem by constraining the numerator (output) of the efficiency ratio to be equal to one and minimizing the weighted input [30]. The model will be solved by giving each role-substitutive model in the sample an efficiency score. The model will compute the factor Z needed to reduce the input of each model to a frontier formed by the remaining models and will be efficient if Z equals one. This composite unit provides targets for the inefficient unit and Z represents the maximum inputs in a service specification that maintains current output [30].

The analysis will be conducted following the general guidance by Hollingsworth [1]. Technical efficiency will be determined across a range of outputs based on the "vital signs" and activity data. Role-substitution will be considered inefficient if the optimal value for the linear programming problem is less than one. If the optimal value is equal to one and if positive optimal multipliers exist then the model will be considered efficient. Improvements in efficiency will then be explored by a proportional reduction of inputs. Efficient, indifferent and inefficient models will be identified and related back to the results of Workstream One.

Stochastic frontier modeling will estimate the efficiency function using a frontier regression model. The standard error will then be used to make assessments of how far each role-substitutive model differs from the most efficient use of role-substitution. Estimates of differences between efficiency will be analysed and interpreted.

The results of both the DEA and SFM will be triangulated with the results of Workstream one and interpreted accordingly. Both DEA and SFM are econometric modeling techniques and as such, do not require a formal power calculation. They do not test a statistical hypothesis based on a frequentist approach.

## Discussion

Given the ad hoc approach to dental service organization in the NHS, it is important to determine the most technically efficient model for role-substitution, *a priori*. In dentistry, role-substitution has the potential to increase efficiency and effectiveness in service provision [31] and increase the capacity to care [32], although this may be situation specific [33]. Therefore, it is not only critical to determine the most technically efficient role-substitutive models, it is equally important to explore the values of policy makers and providers to determine the factors affecting the implementation of such innovative designs and how patients would view such a change in service design [1].

This study will be the first in dentistry to examine the technical efficiency of service provision and has been supported by a National Institute for Health Research’s Health Services and Delivery Research grant (11/1025/04). It has the potential to make a specific contribution to the future commissioning of services across the UK and the development of the new NHS dental contract in England and Wales. It will inform professional groups of the most optimum design, a critical issue for practice principals in England and Wales as income will be capped under the proposed prospective payment system. It will enable a framework for innovation to be developed to transform service delivery and will be of direct relevance to policy makers and health service planners.

## Competing interests

The authors declare that they have no competing interests.

## Authors’ contributions

PRB made the initial application to the National Institute of Health Research’s Health Services and Delivery Research’s funding stream. PRB drafted the original manuscript and received additional comments from RMcD, MT and SB. MT provided oversight on aspects relating to Dental Public Health and RMcD and SB provided additional input in the qualitative and econometric sections respectively. All authors read and approved the final manuscript.

## Pre-publication history

The pre-publication history for this paper can be accessed here:

http://www.biomedcentral.com/1472-6831/13/46/prepub

## References

[B1] Hollingsworth B The measurement of efficiency and productivity of health care delivery Health Econ 2008 17 1107 1128 10.1002/hec.1391 18702091

[B2] Steele J Clarke J Wilson T Rooney E NHS dental services in England: an independent review led by Professor Jimmy Steele 2009 London: Department of Health

[B3] Coalition Government Freedom Fairness Responsibility The Coalition: our programme for government 2010 London: HMG 26

[B4] Birch S Kephart G Tomblin-Murphy G O’Brien-Pallas L Alder R MacKenzie A Human resources planning and the production of health: a needs-based analytical framework Can Public Pol 2007 23 S1 S16 10.1097/PHH.0b013e3181b1ec0e 19829233

[B5] Gallagher JE Wilson NH The future dental work-force? Br Dent J 2009 206 195 200 10.1038/sj.bdj.2009.114 19247334

[B6] Farrell MJ The measurement of productive efficiency J Roy Stat Soc 1957 120 253 281 10.2307/2343100

[B7] Hollingsworth B Street A The market for efficiency analysis of health care organisations Health Econ 2006 15 10 1055 1059 10.1002/hec.1169 16991208

[B8] Adult Dental Health Surveyhttps://catalogue.ic.nhs.uk/publications/primary-care/dentistry/adul-dent-heal-surv-firs-rele-2009/adul-dent-heal-surv-firs-rele-2009-rep.pdf

[B9] Birch S The identification of supplier-inducement in a fixed price system of health care provision. The Case of Dentistry in the United Kingdom J Health Econ 1988 7 129 150 10.1016/0167-6296(88)90012-4 10288955

[B10] Brocklehurst P Tickle M Birch S Glenny AM Mertz E Grytten J The effect of different methods of remuneration on the behaviour of primary care dentists Cochrane Database Syst Rev 2012 5 CD009853 10.1002/14651858.CD009853.pub2 PMC6544809 24194456

[B11] Tickle M McDonald R Franklin J Aggarwal VR Milsom K Reeves D Paying for the wrong kind of performance? Financial incentives and behaviour changes in National Health Service dentistry 1992-2009 Comm Dent Oral Epidemiol 2011 39 5 465 73 10.1111/j.1600-0528.2011.00622.x 21668463

[B12] McDonald R Sudeh C-S Tickle M Changes to financial incentives in English dentistry 2006 to 2009: a qualitative study Comm Dent Oral Epidemiol 2012 40 5 468 7 10.1111/j.1600-0528.2012.00687.x 22462814

[B13] Chalkley M Tilley C Young L Bonetti D Clarkson J Incentives for Dentists in Public Service: Evidence from a Natural Experiment JPART 2010 20 i207 i223

[B14] Chalkley M Tilley C Treatment intensity and provider remuneration: dentists in the British National Health Service Health Econ 2006 15 933 946 10.1002/hec.1162 16958081

[B15] Grytten J Models for financing dental services. A review Community Dent Health 2005 22 75 85 15984132

[B16] Gosden T Forland F Kristiansen I Sutton M Leese B Giuffrida A Sergison M Pedersen L Capitation, salary, fee-for-service and mixed systems of payment: effects on the behaviour of primary care physicians Cochrane Database Syst Rev 2000 3 CD002215 10908531 10.1002/14651858.CD002215 PMC9879313

[B17] Laurant M Reeves D Hermens R Braspenning J Grol R Sibbald B Substitution of doctors by nurses in primary care The Cochrane Database of Systematic Reviews 2005 5 CD001271 15846614 10.1002/14651858.CD001271.pub2

[B18] Laurant M Harmsen J Wolersheims H Grol R Faber M Sibbald B The impact of non physician clinicans: do they improve the quality and cost-effectiveness of health care services? Med Care Res Review 2009 66 36S 10.1177/1077558709346277 19880672

[B19] Sibbald B Shen J McBride A Changing the skill-mix of the health care workforce J Health Services Res Policy 2004 9 Suppl 1 28 38 10.1258/135581904322724112 15006226

[B20] Richardson MSC Identifying, evaluating and implementing cost-effective skill mix J Nurse Management 1999 5 265 70 10.1046/j.1365-2834.1999.00137.x 10786545

[B21] Braun V Clarke V Using thematic analysis in psychology Qual Res in Psych 2006 3 77 101 10.1191/1478088706qp063oa

[B22] Ryan M Scott DA Reeves C Eliciting public preferences for healthcare: a systematic review of techniques Health Technol Assess 2001 5 5 1 186 11262422 10.3310/hta5050

[B23] Jacobs R Alternative Methods to Examine Hospital Efficiency: Data Envelopment Analysis and Stochastic Frontier Analysis Health Care Manag Sci 2001 4 2 103 15 10.1023/A:1011453526849 11393739

[B24] Jacobs R Smith PC Street A Measuring Efficiency in Health Care 2006 Cambridge: Cambridge University Press

[B25] Charnes A Cooper WW Rhodes E Measuring the efficiency of decision making units Eur J Operation Res 1978 2 429 444 10.1016/0377-2217(78)90138-8

[B26] Wagstaff A Estimating efficiency in the hospital sector: A comparison of three statistical cost frontier models Appl Econ 1989 21 659 72 10.1080/758524897

[B27] Skinner J What do stochastic frontier cost functions tell us about inefficiency? J Health Econ 1994 13 323 28 10.1016/0167-6296(94)90031-0 10138858

[B28] Hollingsworth B Peacock SJ Efficiency measurement in health and health care 2008 Oxon: Routledge

[B29] Hollingsworth B Parkin D Developing efficiency measures for use in the NHS 1998 Health Economics Group: A report to the NHS Executive Northern & Yorkshire R&D Directorate. Newcastle

[B30] Parkin D Hollingsworth B Measuring production efficiency of acute hospitals in Scotland, 1991-4: Validity issues in data envelopment analysis Appl Econ 1997 29 1 4 10.1080/000368497327335

[B31] Edelstein BL Examining Whether Dental Therapists Constitute a Disruptive Innovation in US Dentistry Am J Public Health 2011 10 1 5 10.2105/AJPH.2011.300235 PMC3222362 21852623

[B32] Mertz E Glassman P Alternative practice dental hygiene in California: past, present, and future J Calif Dent Assoc 2011 39 1 37 46 21337961 PMC3325901

[B33] Harris RV Sun N Dental practitioner concepts of efficiency related to the use of dental therapists Comm Dent Oral Epidemiol 2012 40 247 256 10.1111/j.1600-0528.2012.00670.x 22324393

